# Specific detection of genetically modified potatoes containing *asparagine synthetase-1* and *polyphenol oxidase 5* genes derived from potato

**DOI:** 10.1080/21645698.2025.2488085

**Published:** 2025-04-07

**Authors:** Sujung Park, Sanggu Lee, Soo-In Sohn, Taesung Park, Kongsik Shin

**Affiliations:** a Department of Agricultural Biotechnology, National Institute of Agricultural Sciences, Rural Development Administration, Jeonju, Korea; b Department of Agricultural Biology, National Institute of Agricultural Sciences, Rural Development Administration, Jeonju, Korea

**Keywords:** Asn1 gene, genetically modified potatoes, GMO detection, monitoring, polymerase chain reaction

## Abstract

Several genetically modified (GM) potatoes have been developed by introducing endogenous genes derived from potatoes, such as *asparagine synthetase-1* (*Asn1*) and *polyphenol oxidase 5* (*Ppo5*), to improve quality. Therefore, it is difficult to distinguish between GM and non-GM potatoes. In this study, we developed a sequence-specific polymerase chain reaction (PCR) detection method to identify innate and inserted genes. We designed four *Asn1* gene-specific primers and eight construct-specific detection primers to evaluate GM potatoes (E12, X17, and Y9) and non-GM crops. Consequently, PCR products corresponding to the original endogenous potato genes and the inserted genes were clearly distinguished and simultaneously identified. In addition, the PCR method demonstrated sufficient sensitivity to identify GM content at levels as low as 0.5%. Thus, this study offers an effective detection method for monitoring or screening approved and unapproved GM potato events using *Asn1* and *Ppo5* transgenes in foods and feeds.

## 1. Introduction

Potatoes (*Solanum tuberosum*) are the fourth most important food crop in the world after rice, wheat, and maize and are regularly consumed by billions of people. They are a low-fat and high-fiber source of healthy carbohydrates and are rich in vitamins and nutrients. In addition, they provide approximately 70% of the proteins found in eggs or milk and generate fewer greenhouse gas emissions than other crops.^1^ Generally, potatoes can be grown in different climates and are easy to cultivate. Globally, 375 million tons of potatoes were produced in an area of 17.8 million hectares in 2022. China (95.6 million tons) and India (56.2 million tons) were the main potato-producing countries, followed by Ukraine, Russia, the United States, and Germany.^2^

Over the past 30 years, genetically modified (GM) crops have been developed and commercialized to improve agricultural management, pest control, environmental stress tolerance, and the functional properties of crops.^3–6^ According to the International Service for the Acquisition of Agri-biotech Applications (ISAAA), as of 2024, 32 crops and 614 events have been approved for the assessment of the safety of GM crops worldwide.^7^ To date, the Korean Ministry of Food and Drug Safety has authorized 189 GM events of six GM crops, including soybean, maize, cotton, canola, alfalfa, and sugar beet, for food use.^8^

Notably, the commercialization of the first insect-resistant GM potato, the New Leaf potato, approved as a food in 1995, was discontinued.^9^ However, a range of traits are now being introduced into potatoes, including insect and disease resistance as well as quality modifications. The ISAAA database indicates that 52 GM potato events were developed and commercialized by 2024.^7^

Furthermore, different functionally improved GM potatoes have been developed to solve various problems that arise when using potatoes as food. For example, potatoes produce acrylamide when heated at high temperatures, and this can increase the risk of carcinogenesis in all age groups.^10^ In addition, potatoes are susceptible to external damage, leading to the development of black spots. *Asparagine synthetase-1* (*Asn1*) gene contributes to the synthesis of asparagine, which reacts with reducing sugars in the Maillard reaction to produce acrylamide. *Polyphenol oxidase 5* (*Ppo5*) is an oxidative enzyme that responds to plant stress by oxidizing phenolics to produce highly reactive quinones that interact with other materials to form brown pigments that contribute to black rot.^11^ GM potato events that lower the acrylamide levels and prevent black spots have been developed to address these challenges.^12^ Notably, as of 2024, 17 GM potato events (E12, E24, E56, F10, F37, G11, Gen2-Z6, H37, H50, J3, J55, J78, V11, W8, X17, Y9, and Z6) have been developed by silencing the *Asn1* and *Ppo5* genes using RNA interference to improve the quality of potatoes.^1,7^

GM potatoes are distributed globally except in Korea, because they are not approved for use as food or feed in Korea.^8^ Korea and many other countries manage unauthorized genetically modified organisms (GMOs) under zero-tolerance or low-level presence (LLP) policies.^13,14^ In addition, as the possibility of unintentional release into the environment increases, rapid and accurate monitoring and detection methods are required to manage unauthorized GMOs. Furthermore, effective detection methods are required to identify GMOs.

Polymerase chain reaction (PCR) is an effective tool for rapidly identifying GMOs in raw and processed materials.^15–17^ Several studies have identified GM potatoes using PCR by targeting relevant genes or DNA sequences introduced into tissues.^6,18–21^ Conventional PCR methods for NewLeaf, NewLeaf Y, NewLeaf Plus, PH05-026-0048, EH92-527-1, and AM04–1020 have been developed,^9,22–25^ and EH92-527-1, AM04–1020, PH05-026-0048, and E12 have been detected using event-specific primers.^12,26^ In particular, event-specific PCR has high specificity for identifying only relevant events. However, this method is challenging because the genetic information on the gene-insertion site must be used for each event.^6,27,28^ In contrast, several GM potato events containing *Asn1* and *Ppo5* genes have been commercialized. However, an effective method to specifically identify the foreign gene is yet to be reported.

In this study, we established a PCR method targeting a specific sequence region of the *Asn1* gene to effectively identify unauthorized GM potato events in Korea, such as E12, X17, and Y9, into which a transgenic cassette containing the endogenous potato genes *Asn1* and *Ppo5* were introduced. We aimed to present an effective and reliable PCR method for detecting GM potato events using the *Asn1* and *Ppo5* transgenes.

## 2. Materials and Methods

### 2.1. Sample Preparation

Reference materials for GM potato (E12, X17, Y9, PH05-026-0048, and EH92-527-1), maize (Mon863), soybean (DAS6846–4), and cotton (M15985) were provided by the developers or purchased from the Institute for Reference Materials and Measurement (IRMM, Geel, Belgium). Seeds or leaves of non-GM potatoes, maize, soybeans, cotton, canola, rice, alfalfa, sweet beets, and tomatoes were purchased from the feed market in Korea.

### 2.2. DNA Extraction

The leaves or seeds of the crops were powdered using liquid nitrogen and a mortar and pestle. Genomic DNA was extracted using a DNeasy Plant mini kit (Qiagen, Hilden, Germany) according to the manufacturer’s instructions. The concentration and purity of the DNA were measured using an ultraviolet spectrophotometer (Thermo Nanodrop 1000, Thermo Fisher Scientific, Waltham, MA, USA). The extracted DNA was adjusted to a concentration of 50 ng/µL for PCR at a 260 nm/280 nm ratio of over 1.8.

### 2.3. Oligonucleotide Primers

**Figure 1.**
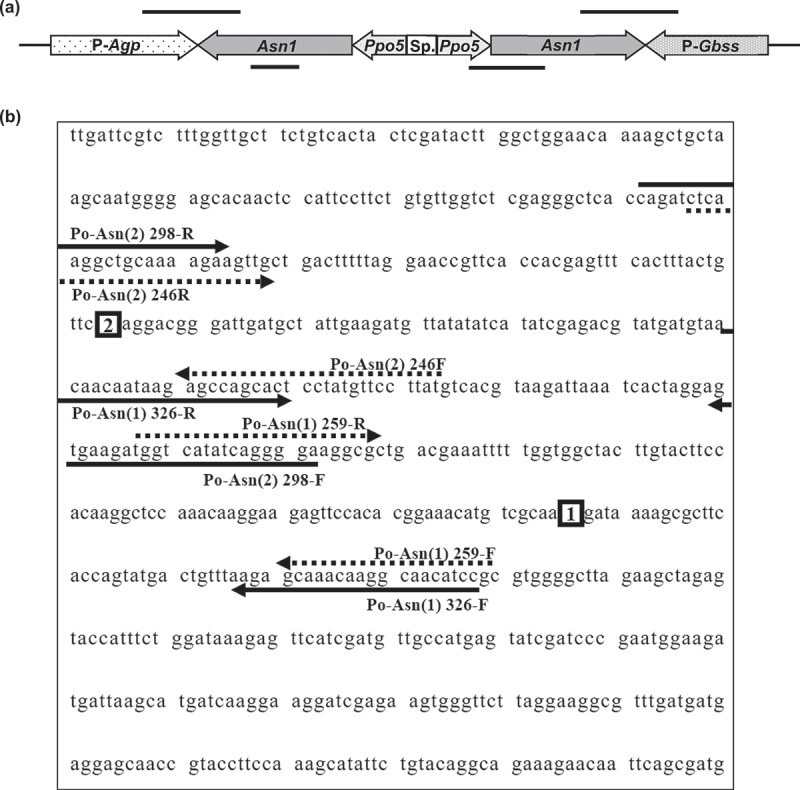
Schematic diagrams of the detection primers and sequences of the *Asn1* transformation cassettes introduced into GM potatoes. This diagram represents the structure of the inserted T-DNA and indicates the four regions detected by primers (a). Boxes 1 and 2 represent regions of the internal sequence of the *Asn1* gene excluded from the inserted gene (b). The arrows below indicate the primers’ detection sites for the foreign *Asn1*. P*-Agp*, promoter of the *ADP glucose pyrophosphorylase* gene; *Asn1*, *asparagine synthetase-1*; *Ppo5*, *polyphenol oxidase 5*; P*-Gbss*, promoter of the *granule-bound starch synthase* gene; *Sp*., spacer.

We developed four *Asn1* gene-specific and eight construct-specific primer pairs to identify the presence or absence of foreign sequences or genetic elements artificially introduced in potato plant events. The *Asn1* detection primers were designed based on two different regions of the transgenic *Asn1* gene (Figure 1(b)), which are major intron sequences in *Asn1* mRNA and have not been introduced into potato. Furthermore, as shown in the T-DNA, the primers were developed to detect the transgenic constructs of *Asn1*, *Ppo5*, adenosine diphosphate-glucose phosphorylase promoter gene (*P-Agp*), and granule-bound starch synthase promoter gene (*P-Gbss*) (Figure 1). The transgenic constructs of GM potato events, such as E12, were verified using the Biosafety Clearing-House site (https://bch.cbd.int/en/). The genetic information of *Asn1* (GenBank Accession Number XM_049553592), *Ppo5* (No. HM363754), *P-Agp* (No. HM363752), and *P-Gbss* (No. HM363755) were obtained from the National Center for Biotechnology Information site (https://www.ncbi.nlm.hih.gov). Nucleotide primers were designed using the primer3 version 0.4.0 (https://bioinfo.ut.ee/primer3-0.4.0/). All primers were synthesized by Bioneer (Daejeon, Korea).

### 2.4. PCR Conditions

PCR was performed in a thermal cycler (Tprofessional Thermocycler; Biometra GmbH, Göttingen, Germany). The reaction mixture consisted of 1 µL of 50 ng/µl DNA, 1 µL of 10 pmole forward primer, 1 µL of 10 pmole reverse primer, and 17 µL of distilled water, all added to the AccuPower® PCR PreMix & Master Mix (Bioneer). The PCR conditions were as follows: initial denaturation at 95°C for 7 min, followed by 30 cycles of denaturation at 95°C for 30 s, annealing at 60°C for 40 s, extension at 72°C for 30 s, and a final elongation at 72°C for 5 min. After PCR, the amplified products were run on 2% (w/v) Ultrapure Agarose gel (Invitrogen, Carlsbad, CA, USA) and stained with Safe-Pinky DNA Gel Staining Solution (GenDEPOT, Houston, TX, USA).

### 2.5. Specificity and Sensitivity of PCR Assay

The specificity of the PCR assay method was evaluated using various crops and GM events. Genomic DNA templates of the GM potato event, Y9, containing the introduced *Asn1* gene were used in concentrations ranging from 3% to 0% (w/w) to determine the detection level of the PCR assay method, and sensitivity tests were performed according to the PCR conditions presented above.

## 3. Results and Discussion

### 3.1. Specific Primers for PCR Detection of Foreign Genes

As of 2014, 17 GM potato events containing *Asn1*, *Ppo5*, *p-Agp*, and *p-Gbss*, such as E12, E24, E56, F10, F37, G11, Gen2-Z6, H37, H50, J3, J55, J78, V11, W8, X17, Y9, and Z6 have been commercialized globally.^7^ The genes which make up the transgenic basal cassette shown in Figure 1(a), are all potato-native genes derived from potatoes. In this study, we aimed to establish a specific PCR detection method to identify GM potatoes containing *Asn1* derived from an endogenous potato gene. Since the above GM potato events were transformed using the original potato endogenous genes, it is difficult to detect these inserted genes in a transgenic plant. We examined the region where the sequence of the inserted gene differed from that originally present in the plant to detect the foreign *Asn1* introduced into the potatoes. We identified two regions on the inserted *Asn1* sequences that did not match the potato endogenous gene. These regions are represented in boxes 1 and 2 of Figure 1(b). These regions were sequences excluded from the innate potato endogenous gene. Therefore, PCR primers that can identify potatoes with and without the inserted *Asn1* gene were derived from these regions (Figure 1(a) and Table 1). In addition, various construct-specific PCR primer pairs targeting *Asn1::Ppo5*, *P-Agp::Asn1*, and *P-Gbss::Asn1* were developed to detect GM potato events, such as E12, X17, and Y9 (Table 1) based on the basic *Asn1*-transformation cassette shown in Figure 1(a).

**Table 1.** Oligonucleotide primers used in this study.

Target gene	Primer name	Sequence (5′-3′)	Size* (bp)	Size of insert** (bp)
***Asn1***	Po-Asn(1)259-F	cggatgttgccttgtttgct	259	151
	Po-Asn(1)259-R	ggtcatatcaggggaaggcg		
	Po-Asn(1)326-F	ggatgttgccttgtttgctct	326	219
	Po-Asn(1)326-R	acaacaataagagccagcact		
	Po-Asn(2)246-F	ataaggaacataggagtgctggc	246	159
	Po-Asn(2)246-R	tctcaaggctgcaaaagaagttg		
	Po-Asn(2)298-F	tcccctgatatgaccatcttcac	298	211
	Po-Asn(2)298-R	cagatctcaaggctgcaaaagaa		
***Asn1/Ppo5***	Po-Asn(1)259-F	cggatgttgccttgtttgct		474
	Ppoop242-R	tctgctgagattacactttgatgga		
	Po-Asn(1)326-F	ggatgttgccttgtttgctct		473
	Ppoop242-R	tctgctgagattacactttgatgga		
	Po-Asn(2)246-F	ataaggaacataggagtgctggc		289
	Ppoop242-R	tctgctgagattacactttgatgga		
	Po-Asn(2)298-F	tcccctgatatgaccatcttcac		337
	Ppoop242-R	tctgctgagattacactttgatgga		
***P-Agp/Asn1***	Agp02-F	ggtcctctcgtaaattccgaca		417
	Po-Asn(1)326-R	acaacaataagagccagcact		
	Agp01-F	agacgtgtgtaccaatcatacct		565
	Po-Asn(2)298-R	cagatctcaaggctgcaaaagaa		
***P-Gbss/Asn1***	Gbss02-F	ggcacctcctcattctcaca		591
	Po-Asn(1)259-R	ggtcatatcaggggaaggcg		
	Gbss01-F	actcactcacacagctcaacaa		568
	Po-Asn(1)259-R	ggtcatatcaggggaaggcg		

*Size indicates the band size of the endogenous gene in the potato.

**The size of the insert represents the band size of the inserted gene region.

In terms of regulatory management, monitoring GM crops is very important, and numerous related studies are focused on monitoring methods for foods and feeds.^29–31^ Of the various methods for monitoring GM crops, the DNA-based approach is the most reliable and currently in general use. In addition, unlike other materials, DNA samples can be prepared and maintained stably, making it the most suitable method for PCR detection.^32–34^ Molecular methods based on PCR are used to determine the presence of small quantities of foreign DNA or transgene inserts in GMOs. PCR-based methods for monitoring and screening GMOs generally utilize event-, gene-, and construct-specific detection methods.^6,28^

Event-specific PCR can effectively detect GMOs using trait-specific molecules, that is, the junction sequences for the integration between a transgene construct and the plant genome.^28^ This method is considered inefficient for screening various GM events because event-specific PCR only detects related traits. Song et al. reported a method for detecting GM potato events involving the introduction of the *Asn1* gene as an event-specific PCR method for only E12.^12^ Currently, 17 GM events with the *Asn1* transgenic cassette inserted have been developed, including E12, X17, and Y9. Therefore, a detection method that can efficiently monitor these several traits simultaneously is needed. However, a PCR method capable of detecting all relevant potato events with the *Asn1* cassette is yet to be reported. In this study, we present a specific detection region for the introduced *Asn1* and a construct-specific primer for the transgenes that can efficiently monitor or screen GM potato events, such as E12.

### 3.2. Specificity of PCR Detection for Asn1 Gene

**Figure 2.**
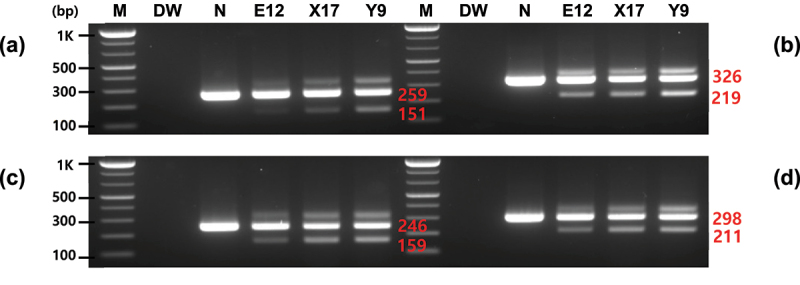
Specificity of the PCR-amplified products using *Asn1* gene-specific primers. PCR was performed by four primer pairs (a, Po-Asn(1)259-F/R; b, Po-Asn(1)326-F/R; c, Po-Asn(2)246-F/R; d, Po-Asn(2)298-F/R). Middle bands represent the endogenous *Asn1* gene (size: 259, 326, 246, and 298 for Po-Asn(1)259-F/R, Po-Asn(1)326-F/R, Po-Asn(2)246-F/R, and Po-Asn(2)298-F/R, respectively) and the lowest bands represent the inserted *Asn1* (size: 151, 219, 159, and 211 for Po-Asn(1)259-F/R, Po-Asn(1)326-F/R, Po-Asn(2)246-F/R, and Po-Asn(2)298-F/R, respectively). Lane M: 1 kb plus DNA ladder, DW: no template, N: non-GM potato, and E12, X17, and Y9: GM potato with foreign *Asn1*.

PCR assays were performed using gene-specific primers developed to detect the introduced *Asn1* gene included in potato events, such as E12, X17, and Y9. Primer pairs were selected based on two specific regions of the inserted *Asn1* sequences. The PCR performed on E12, X17, Y9, and non-GM potatoes resulted in distinct PCR bands that distinguished between GM events and non-GM potatoes (Figure 2). The non-GM potatoes formed only one band, whereas GM events presented two major PCR bands (the lowest and middle bands in E12, X17, and Y9 events depicted in Figure 2). In addition, all events, such as E12, appeared as PCR products of the same form. The primer pairs (Po-Asn(1)259-F/R, Po-Asn(1)326-F/R, Po-Asn(2)246-F/R, and Po-Asn(2)298-F/R) developed in this study showed PCR products of 259, 326, 246, and 298 bp for the original potato endogenous gene(s) and 151, 219, 159, and 211 bp for the introduced *Asn1* gene with the expected sizes. In the E12, X17, and Y9 events depicted in Figure 2, in addition to the two main bands (lowest and middle), an upper band was also observed, indicating that the *Asn1* gene was introduced in the reverse direction as seen in the transformed T-DNA of Figure 1, and thus the base sequence between the *Asn1* on both sides was PCR amplified to form the upper band.

**Figure 3.**
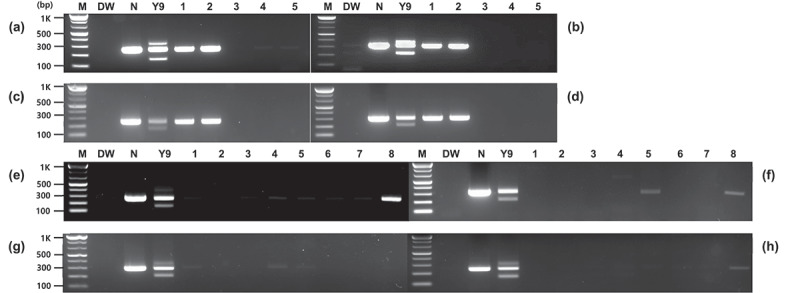
Specificity of the *Asn1* gene-specific primers for several GM and non-GM crops. PCR was performed using four primer pairs (a and e, Po-Asn(1)259-F/R; b and f, Po-Asn(1)326-F/R; c and g, Po-Asn(2)246-F/R; d and h, Po-Asn(2)298-F/R). The lowest bands indicate the foreign *Asn1* (size: 151, 219, 159, and 211 for Po-Asn(1)259-F/R, Po-Asn(1)326-F/R, Po-Asn(2)246-F/R, and Po-Asn(2)298-F/R, respectively). Lane M: 1 kb plus DNA ladder, DW: no template, N: Non-GM potato, Y9: GM potato, in a, b, c and d, 1–5: GM potato PH05-026-0048, GM potato EH92-527-1, GM maize M863, GM soybean DAS 6846–4, and GM cotton M15985, in e, f, g and h, 1–8 (non-GM crops): maize, soybean, cotton, canola, rice, alfalfa, sugar beet, and tomato, respectively. Y9 is one of the major GM potato events containing the *Asn1* cassette.

PCR assays were performed on several GM crops, including GM potatoes, to investigate the detection specificity of *Asn1* gene-specific primers. Unlike Y9 using a cis-regulatory module (CRM), only PCR-amplified products were formed for the endogenous genes in other GM potato events (PH05-026-0048 and EH92-527-1) that do not contain the *Asn1* gene, as in non-GM potatoes. No PCR products were confirmed in other major GM crops, including maize (M863), soybean (DAS6846–4), and cotton (M59857) (Figure 3a–d). In addition, the specificity of the *Asn1-*specific primers was investigated in several non-GM crops. This experiment confirmed that the major crops did not form any PCR products related to the foreign *Asn1*. However, a light band was observed at the endogenous gene location in some crops, especially tomatoes, and this is thought to be due to its genus, which is closely related to that of potato. These results indicate that the four primer pairs developed here could specifically detect the inserted *Asn1* gene in potatoes, allowing for the distinction between GM potatoes and those that naturally contain the gene.

The PCR products of the introduced *Asn1* gene were amplified from regions where the intron sequences that are unnecessary for gene expression were excluded from the original potato endogenous gene. Consequently, the PCR band of the introduced gene could be clearly identified at the same time with a smaller size than that of the original endogenous sequences. When the endogenous gene of a crop is transformed into the same crop, it is difficult to detect the inserted gene. However, transgenes can be distinguished using the deleted or added sequences of the inserted gene. A detection method using the intron region of endogenous genes has been developed for GM maize, such as GA21, containing the herbicide resistance *mEPSPS* gene derived from maize,^35^ and the PCR products of the original endogenous and inserted genes simultaneously appeared in different sizes, similar to this study’s results.

### 3.3. PCR Analysis of Introduced Components

**Figure 4.**
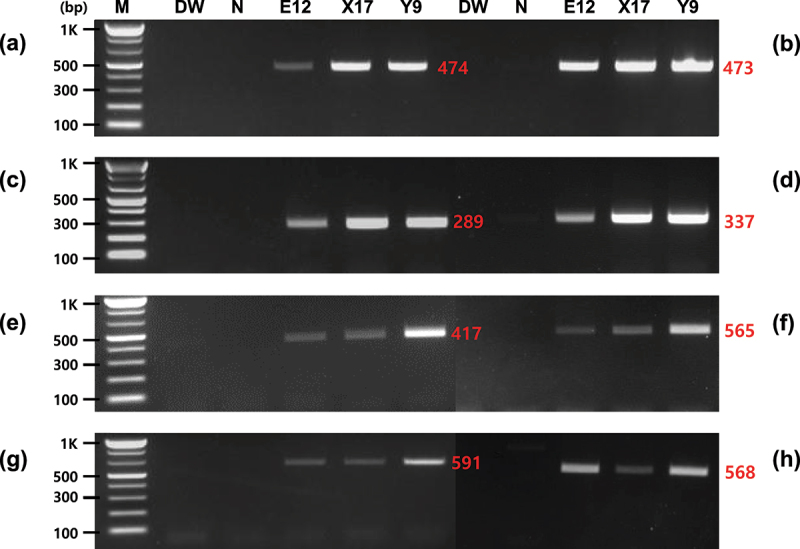
Specificity of the construct-specific primers for GM potatoes containing the *Asn1* cassette. PCR was conducted to detect the structural *Asn1::Ppo5*, *p-Agp::Asn1*, and P*-Gbss::Asn1* regions of the inserted cassette, using eight primer pairs (a, Po-Asn(1)259-F/Ppoop242-R; b, Po-Asn(1)326-F/Ppoop242-R; c, Po-Asn(2)246-F/Ppoop242-R; d, Po-Asn(2)298-F/Ppoop242-R; e, Agp02-F/Po-Asn(1)326-R; f, Agp01-F/Po-Asn(2)298-R; g, Gbss02-F/Po-Asn(1)259-R; h, Gbss01-F/Po-Asn(1)259-R). Lane M: 1 kb plus DNA ladder, DW: no template, N: non-GM potato, E12, X17, and Y9: GM potato events containing the *Asn1* cassette.

Various construct-specific primers were developed based on the *Asn1* transformation cassette introduced in the GM potato events E12, X17, and Y9 to enhance the monitoring and detection method of inserted components. Based on the components constituting the *Asn1* cassette, primer pairs for detecting *Asnl* and *Ppo5* (*Asn1::Ppo5*), *P-Agp* and *Asn1* (*Asn1*::P-*Agp*), and *P-Gbss* and *Asn1* (*Asn1*::P-*Gbss*) were derived, and PCR analysis was carried out using these primers. The PCR results using the construct-specific primers for the three regions on the *Asn1* cassette formed distinct and accurate PCR products for GM potato events E12, X17, and Y9 used as CRMs. In contrast, no amplified products were generated for non-GM potatoes without the *Asn1* cassette (Figure 4).

In GMOs containing the same construct, construct-specific PCR detection was based on DNA sequences in which the two transgenes were adjacent to each other. In addition, many construct-specific PCR assays have been reported, including *p-35S-cry1Ac*, *p-35S-UidA*, and *ctp2-cp4epsps*.^36–38^ Kamle et al. stated that the construct-specific method is not suitable for detecting a single GMO trait, and it is an unreliable and simple method for screening GMOs in GM crops.^28^ However, many GM potato events with the same *Asn1* construct have been developed, and since all transgenes are derived from potato plants, a rapid and simple method for GMO identification is needed. These results are expected to provide an effective and broad screening method for the safety management of approved and unapproved GM events containing the *Asn1* gene.

### 3.4. Sensitivity of PCR Detection of the Asn1 Gene

**Figure 5.**
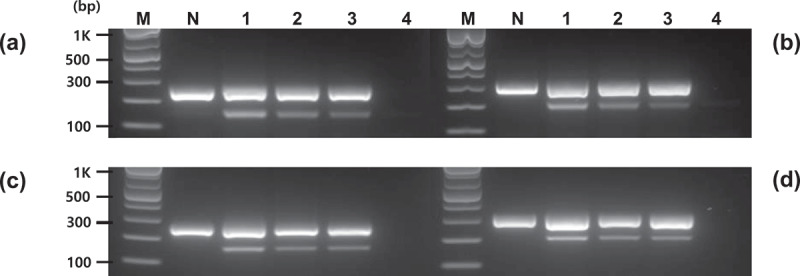
Sensitivity analysis of the *Asn1* gene-specific PCR. PCR sensitivity was performed using four primer pairs (a, Po-Asn(1)259-F/R; b, Po-Asn(1)326-F/R; c, Po-Asn(2)246-F/R; d, Po-Asn(2)298-F/R). Lane M: 1 kb plus DNA ladder, N: non-GM potato, 1–3: 3%, 1%, and 0.5% (w/w), respectively for Y9 potato event, 4: no template.

**Figure 6.**
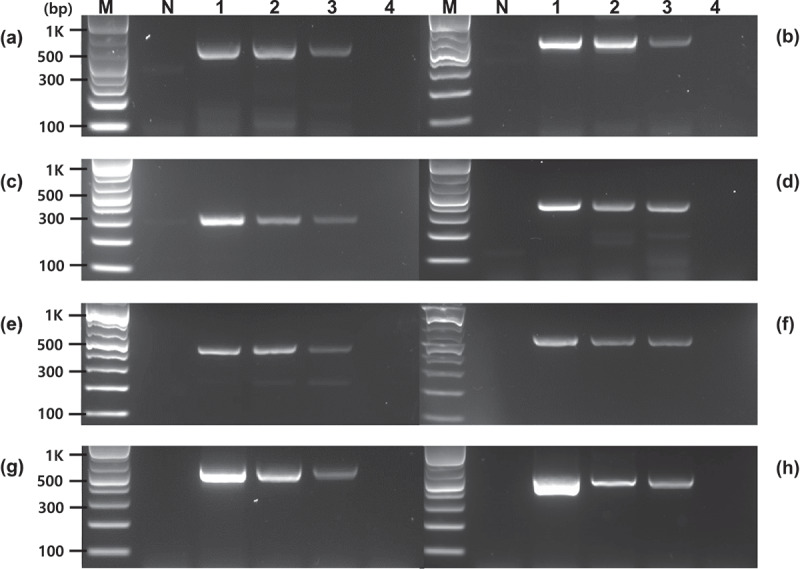
Sensitivity analysis of the construct-specific PCR. PCR sensitivity was analyzed using eight primer pairs (a, Po-Asn(1)259-F/Ppoop242-R; b, Po-Asn(1)326-F/Ppoop242-R; c, Po-Asn(2)246-F/Ppoop242-R; d, Po-Asn(2)298-F/Ppoop242-R; e, Agp02-F/Po-Asn(1)326-R; f, Agp01-F/Po-Asn(2)298-R; g, Gbss02-F/Po-Asn(1)259-R; h, Gbss01-F/Po-Asn(1)259-R). Lane M: 1 kb plus DNA ladder, N: non-GM potato, 1–3: 3%, 1%, and 0.5% (w/w), respectively, for Y9 potato event, 4: no template.

PCR assays were carried out with gDNA samples from Y9 potato events serially diluted to concentrations ranging from 3 to 0.5% (w/w) per reaction to examine the sensitivity of the *Asn1* gene-specific detection method. After *Asn1*-specific PCR, all reaction solutions were run on 2% agarose gel. For GM potato events involving the foreign *Asn1* gene, all the specific primer pairs were shown to sufficiently identify up to 5% of the target DNAs (Figure 5). These results demonstrate that the *Asn1*-specific detection method developed in this study could distinctly detect GMOs incorporated at concentrations as low as 0.5%. Additionally, the PCR detection sensitivity of construct-specific primers (*Asn1::Ppo5*, *p-Agp::Asn1*, and P*-Gbss::Asn1*) was analyzed based on the transformed T-DNA. As shown in Figure 6, PCR detection sensitivity was clearly confirmed up to 0.5% in all construct-specific primer pairs used in this experiment. This detection level is consistent with the LLP of GMOs in countries that are yet to approve or authorize it.^39^ In Europe, products containing > 0.9% GMO must be labeled as “genetically modified” and managed.^34^

As the number of commercial GM events increases, managing unauthorized GMOs has become important in countries around the world, including Korea. Various methods for detecting DNA introduced into crops are being developed to address this issue of GMO management. Approaches to several GMO screening methods have been conducted to target promoters, terminators, coding genes, and other components of the introduced constructs. Representatively, methods for detecting major factors introduced in GM crops, such as *CMV 35S* promoter, *Nos* terminator, *cry1Ac*, and *cp4epsps*, have been reported.^36,38,40^ In addition, event-specific, multiplex, and real-time PCR methods have been developed for screening GM crops.^12,14,41^ Most of these reports were GMO screening methods targeting general transgenic factors, including *p-35S* and *T-nos*, amongst others. Recently, several GM potato events have been developed by introducing *Asn1* (inherently present in potatoes) into potato plants. Since the potato was transformed with components composed of the original potato endogenous genes, a GMO screening method for the related introduced factors is yet to be reported. In this paper, we describe a specific, sensitive, and reliable screening method for identifying several GM potato events containing the *Asn1* construct.

## 4. Conclusions

We developed a PCR method to detect GM potato events such as E12, X17, and Y9, which contained *Asn1*, a potato endogenous gene. PCR markers were derived from regions where the original endogenous gene sequence of potato and the inserted gene sequence differed to detect the introduced *Asn1*. The primers developed in this study clearly distinguished and detected the introduced *Asn1* gene. This PCR assay also accurately identified potato events with foreign insertion of *Asn1* in other GM and non-GM crops. In addition, the sensitivity of this method was sufficient to monitor the presence of GM potatoes containing inserted *Asn1* in foods and feeds. Therefore, this PCR detection method could serve as a specific and sensitive method to identify and trace GM potato events involving the inserted *Asn1* in the safety management of approved and unapproved GMOs.

## Data Availability

Data will be made available on request.
